# Malondialdehyde concentrations in obsessive–compulsive disorder: a systematic review and meta-analysis

**DOI:** 10.1186/s12991-021-00354-2

**Published:** 2021-06-16

**Authors:** Amir Hossein Mohammadi, Ebrahim Balandeh, Alireza Milajerdi

**Affiliations:** 1 Student Research Committee, Kashan University of Medical Sciences, Kashan, Iran; 2 Research Center for Biochemistry and Nutrition in Metabolic Diseases, Institute for Basic Sciences, Kashan University of Medical Sciences (KAUMS), Kashan, Iran; 3 Department of Clinical Psychology, School of Medicine, Kashan University of Medical Sciences, Kashan, Iran

**Keywords:** Oxidative stress, Malondialdehyde, Obsessive–compulsive disorder, OCD, Meta-analysis

## Abstract

**Background:**

This meta-analysis aimed to investigate serum and plasma malondialdehyde (MDA) levels in patients with obsessive–compulsive disorder (OCD) in comparison to healthy controls.

**Methods:**

Following the PRISMA protocol, we searched for the relevant studies through the databases of Scopus, PubMed, Google Scholar, and web of science until September 2019 with no time restriction. Overall, nine studies were included in the current meta-analysis. Data were pooled using a random-effects model; in addition, standard mean difference (SMD) and/or weight mean difference (WMD) was calculated. Cochran’s Q test and I-square (I^2^) statistics were used to evaluate between-study heterogeneity. The Newcastle–Ottawa scale (NOS) was used to evaluate the quality of the included studies. Statistical analyses were done using the STATA version 14.

**Results:**

Our systematic review included nine case–control studies (including 367 cases and 337 controls). Pooling findings from these studies showed a significantly higher MDA level in OCD patient compared to control groups (SMD = 1.62; 95% CI [0.53, 2.72]; I^2^ = 96.9%; Pheterogeneity (Ph) < 0.001). This finding remained unchanged among studies which reported MDA in the same unit (WMD = 1.93; 95% CI [0.27, 3.59]; I^2^ = 99.2%; Ph < 0.001). Subgroup analysis by the study location and sample size revealed findings that were also significant.

**Conclusion:**

We found that MDA levels are higher in OCD patients than healthy controls. This finding highlights the importance of inflammatory responses in OCD patients that should be considered for future investigations. Further studies are recommended to expand current knowledge on this issue.

## Introduction

Obsessive–compulsive disorder (OCD) is characterized by intrusive, unwanted, and disturbing thoughts, images, and urges (obsessions) that elicit significant distress and repetitive behavioral or mental compulsions [1]. Such compulsions may lead to a temporary reduction in distress [2]. About 0.5–3% of people suffer from this disorder [3, 4] which influences social relations, quality of life, and occupational functioning [5–7]. Moreover, family members of the patients who live with him/her are also affected by the disorder [8].

Oxidative stress (OXS), resulting from imbalances between the generation of oxidative free radicals in the body and neutralizing antioxidants, plays an important role in the pathophysiology of OCD [9]. OXS can cause several damages to the brain, including disruption of the membrane integrity, damages caused by the peroxidation of lipids, proteins, and nucleic acids, and changes in natural neuronal apoptosis [10]. Free oxygen radicals have been recently acknowledged as great risk factors in the pathogenesis of several diseases, including neuropsychiatric disorders [11, 12]. OXS also influences the genes responsible for various psychiatric disorders, including obsessive–compulsive disorder and other anxiety disorders [9, 13]. Failure to regulate endogenous oxidative processes leads to brain insult, which may be implicated in various psychiatric disorders [14]. OXS may damage, modify, and denature the chemical structure of fatty acids [15]. Emerging evidence suggests that OXS and inflammation accelerate cellular aging and neuroprogression [16]. The brain is particularly vulnerable to the deleterious effects of OXS. Studies have linked OXS to blood–brain barrier disruptions, altered patterns of neural growth, and changes in brain morphology [17, 18]. This condition can cause psychological stress-related mental illnesses [16] and neurobehavioral disorders [19]. Therefore, the measurement of OXS in patients with OCD is important.

Malondialdehyde (MDA) is a natural product of peroxidation of unsaturated fatty acid with three or more double bonds. MDA concentration is usually measured by the thiobarbituric acid reacting substances test (TBARS), which is known as a simple and non-specific test [20]. MDA measurement is a reliable method and is known as one of the most popular methods for determining the status of OXS in clinical settings [20]. Moreover, MDA has been found in brain centers and its factors play crucial roles in the pathogenesis of neurodegenerative diseases [21]. Earlier studies have measured serum MDA levels in OCD patients compared to healthy controls [22, 23]. Results suggested higher free radical metabolism in OCD and therefore tissue damage due to OXS [22]. However, few studies have summarized their findings to reach a firm conclusion. Although a meta-analysis in 2019 investigated levels of oxidative and nitrosative markers in patients with OCD [24], that study had several limitations [25] including missing some relevant publications [26, 27]. In addition, the authors pooled data with different units in their meta-analysis, possibly leading to invalid results. A similar study by Oliveira et al. also had the same limitations [28]. Oliveira et al. suggested that “Ozdemir et al. was in fact selected for systematic review, but excluded from quantitative analyses, as stated in the PRISMA flow diagram”; however, they referenced Ozdemir et al. in included studies in Maia et al. [24]. Due to these mistakes, reaching a firm conclusion seems impossible. By contrast to the limitations of the prior study, ours had some strengths, namely in our systematic review, we included more studies with controversial findings and also including a sufficient number of studies allowed us to do subgroup analysis, all of which contribute to a more reliable conclusion.

As mentioned above, OXS greatly influences brain function and might influence disease prognosis in patients with OCD. Therefore, the measurement of OXS is crucial for these patients. MDA is a known independent marker of OXS and inflammation that has been frequently measured in such patients. Although several earlier studies have been published about serum levels of MDA in patients with OCD, there is still the need to summarize their findings. Measurement of MDA in patients with OCD compared to healthy controls gives us important information about OXS in these patients and the accuracy of MDA measurement for detection of OXS and for expectation of disease prognosis. Therefore, we aimed to conduct current systematic review and meta-analysis to summarize findings from earlier studies in which MDA was measured in OCD patients compared to healthy controls. It should be mentioned that among different markers of OXS, sufficient data were available only for MDA levels.

## Methods

### Search strategy

The present study was performed based on the PRISMA protocol for reporting systematic reviews and meta-analyses [29]. A systematic search was done by two independent investigators (AM and EB) through databases of PubMed, Web of Science, Science Direct, Scopus, and web of science on relevant studies published up to December 2019. Keywords used in our search strategy were: (("Obsessive–compulsive disorder" OR "Obsessive–compulsive disorder*" OR "OCD" OR "Obsessive–compulsive" OR "Obsession" OR "Compulsion" OR "Obsess" OR "Compuls" OR "Obsessive*-compulsive" OR "Obsessive–compulsive*" OR "Obsessive*-compulsive*" OR "Anankastic"[MESH]) AND ("MDA" OR "Malondialdehyde" OR "Malonyldialdehyde" OR "Propanedial")). No restriction was made in terms of publication time or language. Also, the reference lists of relevant papers were reviewed to avoid missing any publication. Data from unpublished studies were not included. If two studies were published on the same set of data, we included only the most complete one [30].

### Inclusion and exclusion criteria

In this meta-analysis, publications with the following criteria were considered to be eligible: (1) studies in which OCD cases were diagnosed according to the Diagnostic and Statistical Manual of Mental Disorders (DSM-III or later edition), or its equivalent in the International Classification of Diseases (ICD); (2) studies that evaluated MDA levels in patients with OCD; (3) studies that recruited healthy subjects as controls; (4) those that reported mean ± standard deviation (SD) MDA level along with 95% confidence intervals. Letters, comments, short communication, reviews, meta-analyses, ecological studies, and animal studies were also excluded.

### Data extraction

Necessary data were extracted from included studies by two independent reviewers. All reported mean ± SD for the MDA level compared with the healthy control group were extracted. We obtained the following information from each study: first author's name, year of publication, country of origin, the age range at study baseline, gender, sample size, study design, number of participants, methods used for assessing MDA levels and OCD, and any adjustments for confounding variables (Table 1).

**Table 1:** General characteristics of included studies

Country (ethnicity)	Control		Case		OCD diagnosis	Method of MDA assay	Sample size (Ca/Co)	Age (Ca/Co)	Gender (Ca/Co)		Severity				SYB	Duration (years)	Match	Quality assessment	Result	Reference
	Mean	SD	Mean	SD					F	M	Mild	Mo	Severe	Vs						
Iraq (Caucasian)	1.15 nmol/ml	1	1.67	3.28	NM	ELISA	(60/30)	18–68 years	43	47	NM	NM	NM	NM	NM	NM	NM	3	↑	Mohamed et al. 2020
India (Asian)	3.85 unit/mg of protein	1.83	1.02	0.5	ICD-10-DCR	Ohkawa	(30/30)	(23.86 ± 6.96/28.56)	(19/19	(11/11)	NM	NM	NM	NM	23.8 ± 1.90	3.9 ± 2.9	Age and gender	6	↑	Shrivtastava et al. 2017
Turkey (Caucasian)	10.32 nmol/ml	1.51	5.13	0.98	DSM IV	Ohkawa	(102/104)	(38.75 ± 10.88/36.39 ± 10.35)	(82/57	(20/47)	NM	NM	NM	NM	NM	11.26 ± 8.56	Age and gender	5	↑	Orhan et al. [34]
Bangladesh (Asian)	5.7 nmol/ml	1.7	4.3	1.2	DSM IV	Satoh	(48/48)	(24.7 ± 4.6/21.3 ± 1)	(6/6)	(42/42)	16	18	14	NM	NM	< 5 30, 5–10 8, > 10 10	Age, gender and socioeconomic status	7	↑	Shohag et al. [22]
India (Asian)	4.89 nmol/ml	0.22	4.07	0.22	DSM IV	Satoh	(20/20)	(29.70 ± 8.35/30.50 ± 9.34)	(0/0)	(20/20)	NM	NM	NM	NM	NM	Least of a year	Age and gender	5	↑	Behl et al. [32]
Turkey (Caucasian)	10.139 nmol/g Hb	8.68	6.032	6.57	DSM IV	Jain	(28/28)	(28.28 ± 5.41/28.85 ± 5.54)	(18/18	(10/10)	5	11	9	3	22.47 ± 5.39	NM	Gender	6	↑	Ozdemir et al. [26]
Turkey (Caucasian)	0.285 μmol/g Hb	0.05	0.198	0.03	DSM IV	Jain	(30/30)	(29.2 ± 6.2/ 29.2 ± 7.2)	(22/21)	(8/9)	NM	NM	NM	NM	NM	NM	Age and gender	6	↑	Ersan et al. [23]
Turkey (Caucasian)	4.15 nmol/ml	0.73	2.5	0.41	DSM IV	Satoh	(34/32)	(25.72 ± 7.04/29.13 ± 9.19)	(22/19)	(12/13)	4	9	18	3	NM	5.35 ± 3.12	Age and gender	6	↑	Kuloglu et al. [33]
India (Asian)	12.63 nmol/ml RBC	2.91	8.84	0.46	DSM IV	Wills	(15/15)	NM	NM	NM	NM	NM	NM	NM	NM	NM	Age and gender	3	↓	Kurup et al. [27]

*Ca* case, *Co* control, *SYB* score of Y-BOCS, *Mo* moderate, *Vs* very severe, *F* female, *M* male, *NM* not mentioned by the authors/in the article

### Quality assessment

The Newcastle Ottawa scale (NOS), designed for observational studies, was used to assess the quality of selected studies [31]. The NOS gives a maximum of ten-point to each study: five for selection, two for comparability, and three for outcome assessment (ten represented the highest quality). Any discrepancies were resolved by discussion. In the current study, those studies with a NOS score of 5 or more were considered high-quality publications (Table 1).

### Statistical analysis

We pooled mean ± SD of MDA levels reported in persons with OCD compared to control groups using a random-effects model. To assess between-study heterogeneity, the Cochrane Q test and I^2^ statistics were used. Between-study heterogeneity was considered significant when I^2^ values were 50% or more. Besides, we conducted subgroup analyses by the predefined criteria to find probable sources of heterogeneity. Statistical analyses were done using STATA version 14 (StataCorp).

## Results

### Characteristics of included studies

**Fig. 1:**
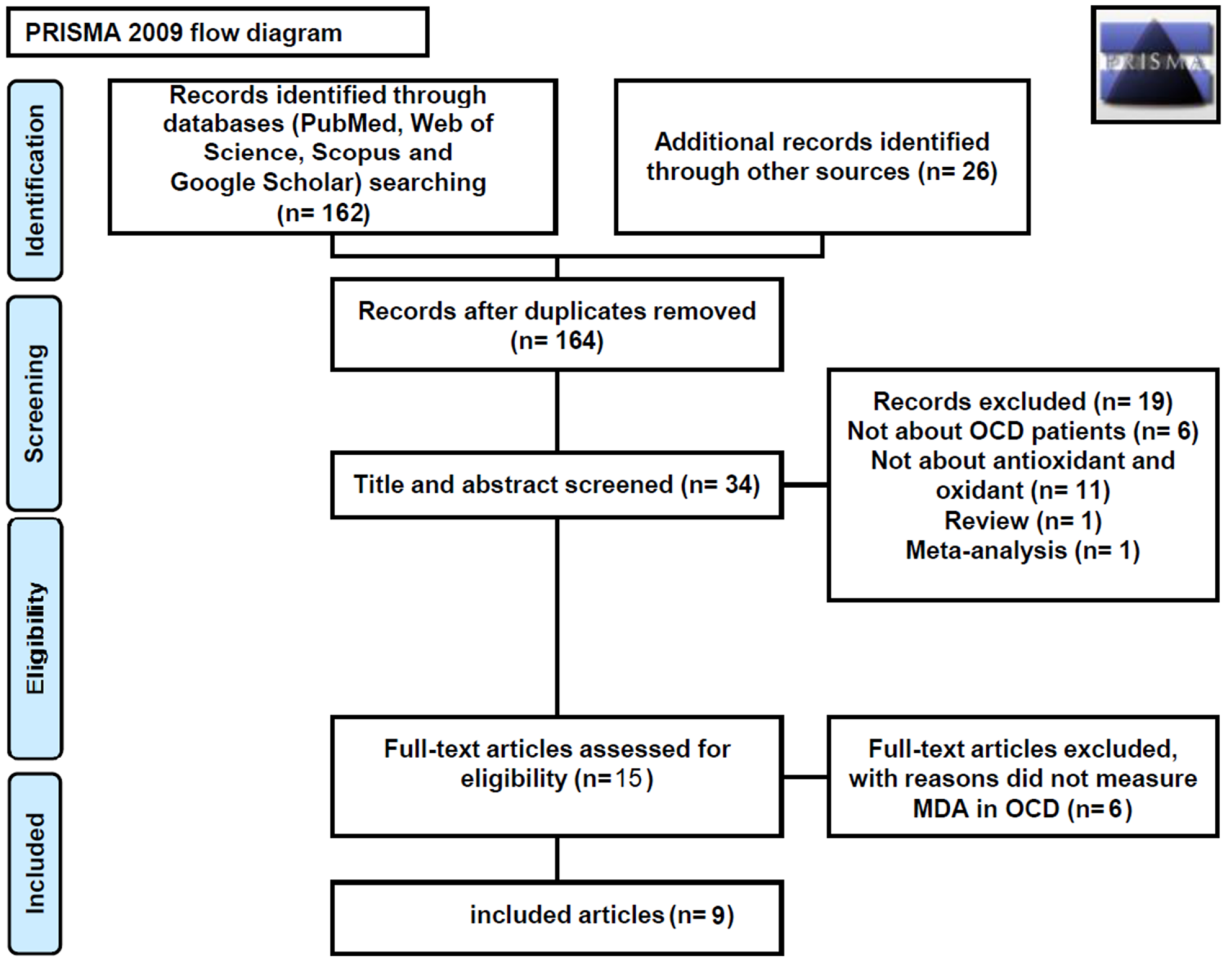
PRISMA flow diagram, flowchart of the number of studies identified and included into the meta-analysis

Our systematic review included nine case–control studies [22, 23, 26, 27, 32–36]. Figure 1 presents a flow diagram for study selection. The characteristics of these studies are shown in Table 1. The sample size of these studies varied from 30 to 206 [total: patient 367 (women 57%, men 43%) and control 337 (women 49% men 51%)]. The mean age of patients was 28.6 years and the mean age of control was 29.13 years. The included studies were published between 2002 and 2019. Four studies were conducted in Turkey [23, 26, 33, 34], three in India [27, 32, 35], one in Bangladesh [22], and Iraq [36]. Among the studies, one was conducted on male subjects [32], the six were conducted on both genders [22, 23, 26, 33–35], and two other studies did not report the gender of the subjects [27, 36]. The mean disease duration ranged from 3.9 to 11.26 years. OCD was diagnosed based on DSM-IV in seven studies [22, 23, 26, 27, 32–34], while one study used ICD-10-DCR [35] and one study did not report the diagnostic method [36]. In addition, two studies used Y-BOCS to assess the severity of OCD [26, 35]. Moreover, few studies did not report the Y-BOCS outcome [22, 23, 27, 32–34, 36]. MDA level was measured based on the method developed by Satoh [22, 32, 33], Ohkawa [34, 35], Jain [23, 26], Wills [27] and one study used ELISA method [36]. Not having enough access to sufficient data, we could not unify all studies.

### Results from meta-analysis

**Fig. 2:**
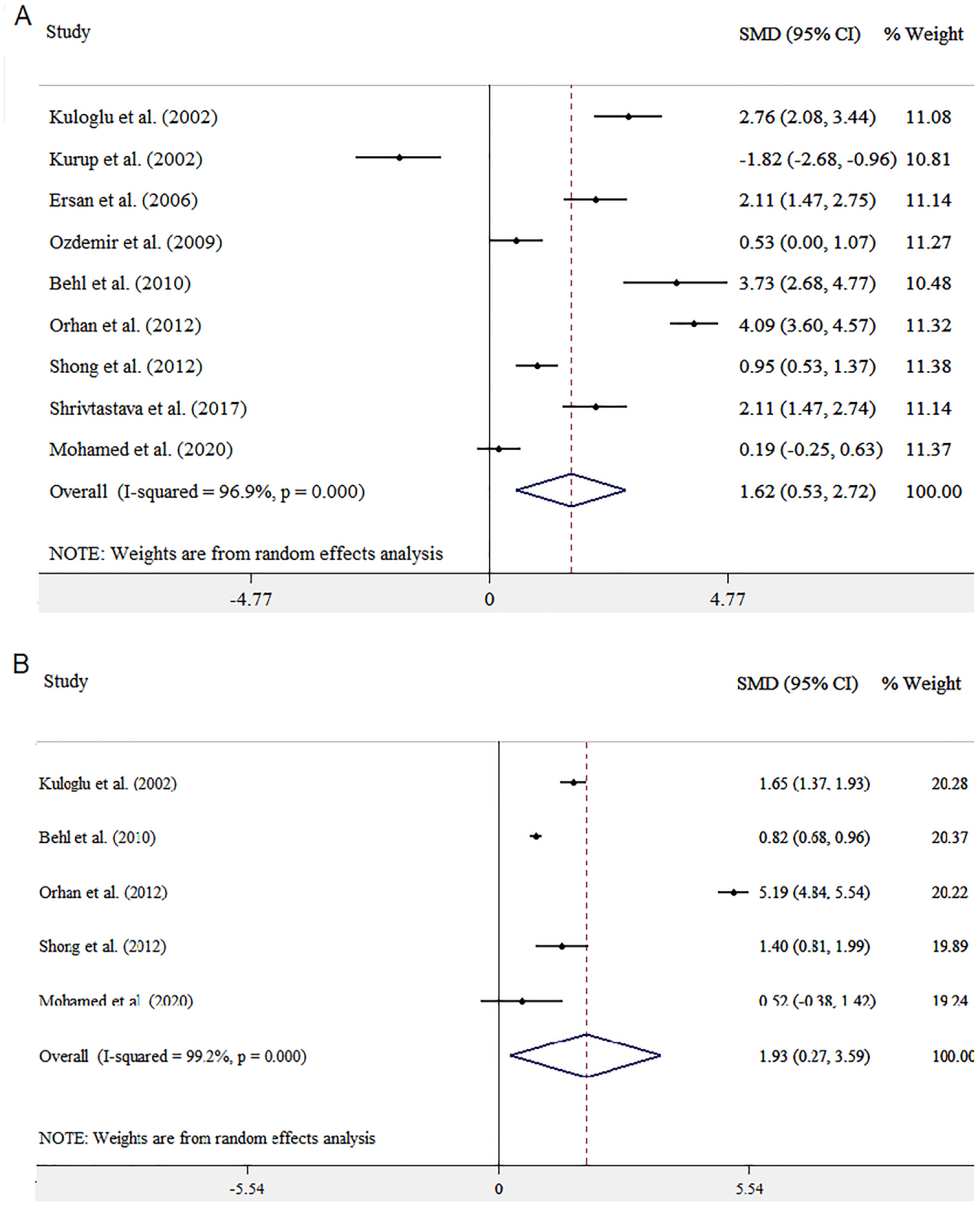
Forest plot displaying mean difference and 95% confidence intervals (CI) for the MDA concentration **A** different unit, and **B** same unit, horizontal lines represent 95% CI. Diamonds represent pooled estimates from random-effects analysis

The results of overall and stratified analysis are summarized in Table 2. Pooling data from 9 studies, we found that MDA level was significantly higher in patients with OCD comparing to control groups (SMD = 1.62; 95% CI [0.53, 2.72]; I^2^ = 96.9%; Pheterogeneity (Ph) < 0.001). When we repeated our analysis among those studies that reported MDA levels in same unit, overall finding remained unchanged (WMD = 1.93; 95% CI [0.27, 3.59]; I^2^ = 99.2%; Ph < 0.001) (Fig. 2). Between-study heterogeneity was high among the included studies. We performed subgroup meta-analysis based on participants’ country (Turkey and other countries) and study sample size (up to 60 and more than 60) to find possible sources of heterogeneity. Similar findings were also reached in all subgroups (Table 2).

**Table 2:** Malondialdehyde concentration in relation to risk of obsessive-compulsive disorder by random effect model

Malondialdehyde			N studies	SMD	WMD	95% CI	I^2^ (%)	*P*h
Different unit	Overall		9	1.62		[0.53, 2.72]	96.9	< 0.001
	Country	Turkey	4	2.46		[2.18, 2.75]	96.9	< 0.001
		Other	5	0.81		[0.55, 21.06]	95.6	< 0.001
	Sample size	≤ 60	5	1.24		[0.93, 1.54]	95.6	< 0.001
		> 60	4	1.73		[1.49, 1.97]	98.1	< 0.001
Same unit			5		1.93	[0.27, 3.59]	99.2	< 0.001

Ph (P< 0.1 was considered as a significant difference), * SMD* Standardized Mean Difference, * WMD* weighted mean difference

#### Discussion

The current meta-analysis showed that MDA level was higher in OCD patients compared to control groups. This finding is in line with Maia et al. meta-analysis, where the same result was reached [24]. However, that study had numerous methodological limitations [25]. To solve those limitations, we added the missed studies. In addition, we have added more studies. Also, we have evaluated the MDA concentration alone. Jiménez-Fernández et al. (2015) and Liu et al. (2015) reported that the MDA level was higher in patients with depression disorder than controls [37, 38]. Moreover, Flatow et al. (2013) reported higher MDA levels in patients with schizophrenia than controls [39]. Other studies also showed significantly higher lipid peroxidation and nitric oxide in patients with psychological disorders than controls [40, 41]. In contrast with our study, Ranjekar et al. (2003) found no significant differences in serum levels of lipid peroxidation markers between patients with schizophrenic and healthy control [42]. Furthermore, Talarowska et al. (2011) did not find any significant association between plasma MDA levels and depressive disorder [43]. Oxidative markers are unspecific and can be influenced by numerous factors, such as body mass index, age, or smoking status [44–46]. Our subgroup analysis showed no overall change in our findings. However, due to the limited number of included studies in each subgroup, further studies are needed to reach a firm conclusion.

Although the exact mechanism of the association between OXS and OCD has not been clearly understood, some suggestions are made. ATP production in mitochondria by electron transport chain or through the respiratory burst of macronutrients in macrophages and neutrophils produce OXS [47]. Increased production of reactive oxygen species (ROS) overcomes antioxidant systems and causes injuries to the lipids, proteins, including some neurotransmitters (NT), and DNA [41]. Several NT containing dopamine, serotonin, gamma-aminobutyric acid, and glutamate have been shown to have regulatory roles in OCD [34]. Changes in these NT pathways may then increase oxidative reactions in the central nervous system [48]. Previous studies have shown that MDA reduces levels of serotonin in the brain [49]. OCD symptoms also result from some damages in the dopamine receptor-rich basal ganglia [50]. Also, OXS could at first be adaptive through enhancement of NT. However, that prolonged ROS generation could lead to exaggerated neurophysiological responses, with disruption of physiological NT [17] and increase of blood–brain barrier permeability, ultimately promoting neuroinflammation and neuronal death [51]. Furthermore, genetic factors might contribute to pathogenesis and symptoms of OCD [52, 53]. OCD is prevalent among those with mitochondrial dysfunctions induced by genetic disorders [34]. OXS is shown to be high among these subjects.

This meta‐analysis had several limitations. Firstly, the number of the included studies was insufficient to do subgroup analyses. Secondly, in the studies that MDA concentrations were reported as µm per mg of protein or hemoglobin, we needed mean protein or hemoglobin concentrations to convert these values to µm. We asked the first authors or study corresponding authors to send us the necessary data, but no response was received. Thirdly, differences in study location and limited number of participants were other limitations. Fourthly, findings were not adjusted for several important confounding factors, such as dietary intakes, body mass index, and smoking status. Fifthly, studies used different laboratory methods to measure MDA serum levels and diagnose OCD, so this should be concerned as a source of bias. Finally, most included studies were done among a certain population (Turkish).

In conclusion, our study showed that MDA levels in patients with OCD were higher than in healthy controls. Studies have shown that antidepressant agents, which are commonly used in OCD patients, reduce oxidative stress markers in the body [54, 55]. The current study showed that oxidative stress was high in these patients. Therefore, it might be suggested that measurement of oxidative stress markers in these patients will be a treatment biomarker for the future. Further investigations (among different nations) are required to shed light on the role of OXS in OCD initiation and pathogenesis.

## Data Availability

Data and material will be provided upon request.
